# Methylphenidate regulation of osteoclasts in a dose- and sex-dependent manner adversely affects skeletal mechanical integrity

**DOI:** 10.1038/s41598-018-19894-x

**Published:** 2018-01-24

**Authors:** Sardar M. Z. Uddin, Lisa S. Robison, Dennis Fricke, Evan Chernoff, Michael Hadjiargyrou, Panayotis K. Thanos, David E. Komatsu

**Affiliations:** 1 Department of Orthopaedics, Stony Brook University, Stony Brook, NY 11794 USA; 2 Department of Psychology, Stony Brook University, Stony Brook, NY 11794 USA; 3 Research Institute on Addiction, University at Buffalo, Buffalo, NY 14203 USA; 4 Department of Life Sciences, New York Institute of Technology, Old Westbury, NY 11568 USA

## Abstract

Methylphenidate (MP) is the most prescribed psychostimulant for ADHD patients, with clinically demonstrated detrimental effects on bone quality, potentially leading to early onset osteoporosis and higher fracture risk. The underlying mechanism for the effects of MP on bone remains elusive. This study demonstrates that sex- and dose-dependent effects of MP on bone quality and quantity are mediated by osteoclast activity. Four-week-old male and female rats were treated with low and high dose MP for 13 weeks. Bone quality and quantity were analyzed using microCT, mechanical testing, histomorphometry, and TRAP staining. Male and female rat bone marrow-derived osteoclasts were treated in a dose-dependent manner (0–1000 ng/ml) and osteoclast activity was determined at days 5, 7, and 14 using TRAP staining, as well as a pit formation assay at day 18. Animal studies showed a dose- and a sex-dependent decrease in mechanical integrity in femora and increased TRAP staining in MP-treated rats. Primary cultures revealed that MP had direct dose- and sex-dependent effects on osteoclast activity, as seen by increased differentiation, activity, and resorption. This study demonstrates for the first time that osteoclasts are differentially regulated by MP in adolescent male and female rats, resulting in sex-dependent effects on the skeleton.

## Introduction

Attention Deficit Hyperactivity Disorder (ADHD) is a psychiatric disorder associated with hyperactivity, impulsivity, and inattention. Commonly diagnosed in children and resulting in a lifelong prognosis, ADHD affects 1 in 11 Americans, with approximately twice the prevalence in males compared to females^1^. Methylphenidate (MP) is one of the most prescribed medications for ADHD, with >10 million prescriptions per year and a 65–75% clinical effectiveness^2,3^.

MP is generally well tolerated but has been reported to adversely affect skeletal growth. A multimodal treatment study of ADHD in 7–9-year-olds showed 0.9 cm/yr growth suppression over the 14-month study, with an additional 1.03 cm/yr growth suppression during a 10-month follow-up in the MP-treated patients relative to age-matched peers^4^. Even stronger growth suppression of 1.38 cm/yr was reported in the Preschool ADHD Treatment Study (PATS)^5^. A recent dual-energy X-ray absorptiometry (DXA) study of 6489 people aged 8–20 years compared psychostimulant users (>3 months) to non-users and found significant decreases in the lumbar spine (0.83 vs 0.73), femoral neck (0.84 vs 0.77), and total femur (0.91 vs 0.83) bone mineral density (BMD, g/cm^3^), as well as significant decreases in the lumbar spine (10.81 vs 9.07), femoral neck (4.07 vs 3.62), and total femur (29.12 vs 25.51) bone mineral content (BMC, g)^6^. Unfortunately, the investigators did not assess the sex-specific effects of MP on bone quantity.

MP-induced growth repression has also been reported in rats, with some studies showing repression to be independent of food intake^7^. It has also been reported that MP-induced growth repression is transient and recovered within 30 days post-treatment^8^. The skeletal effects of MP are not only limited to growth but have also been shown to impair bone quality and quantity. Komatsu *et al*. showed that adolescent male rats treated with MP demonstrated dose-dependent weight loss after 13 weeks, with a 5–9% decrease in femoral anterior-posterior (AP) diameter^9^. DXA analyses revealed dose-dependent reductions in BMD and BMC. Critically, femoral biomechanical integrity was significantly compromised by MP, with a 20% and 30% decrease in energy to failure in low and high dose MP-treated rats, respectively. The study was the first to demonstrate adverse effects of MP on bone quality and quantity but was limited to male rats. Given the sex-dependent behavioral effects of MP^10,11^, sex-dependent skeletal effects need to be assessed.

While it is now unequivocal that MP can impair skeletal growth and bone quality clinically and in animal models^6,9^, the mechanism by which MP causes these adverse effects remains unclear. Therefore, the objective of this study was to determine the effects of MP on skeletal development in male and female rats and identify the underlying mechanism(s).

## Results

### MP reduces weight gain and increases activity

**Figure 1:**
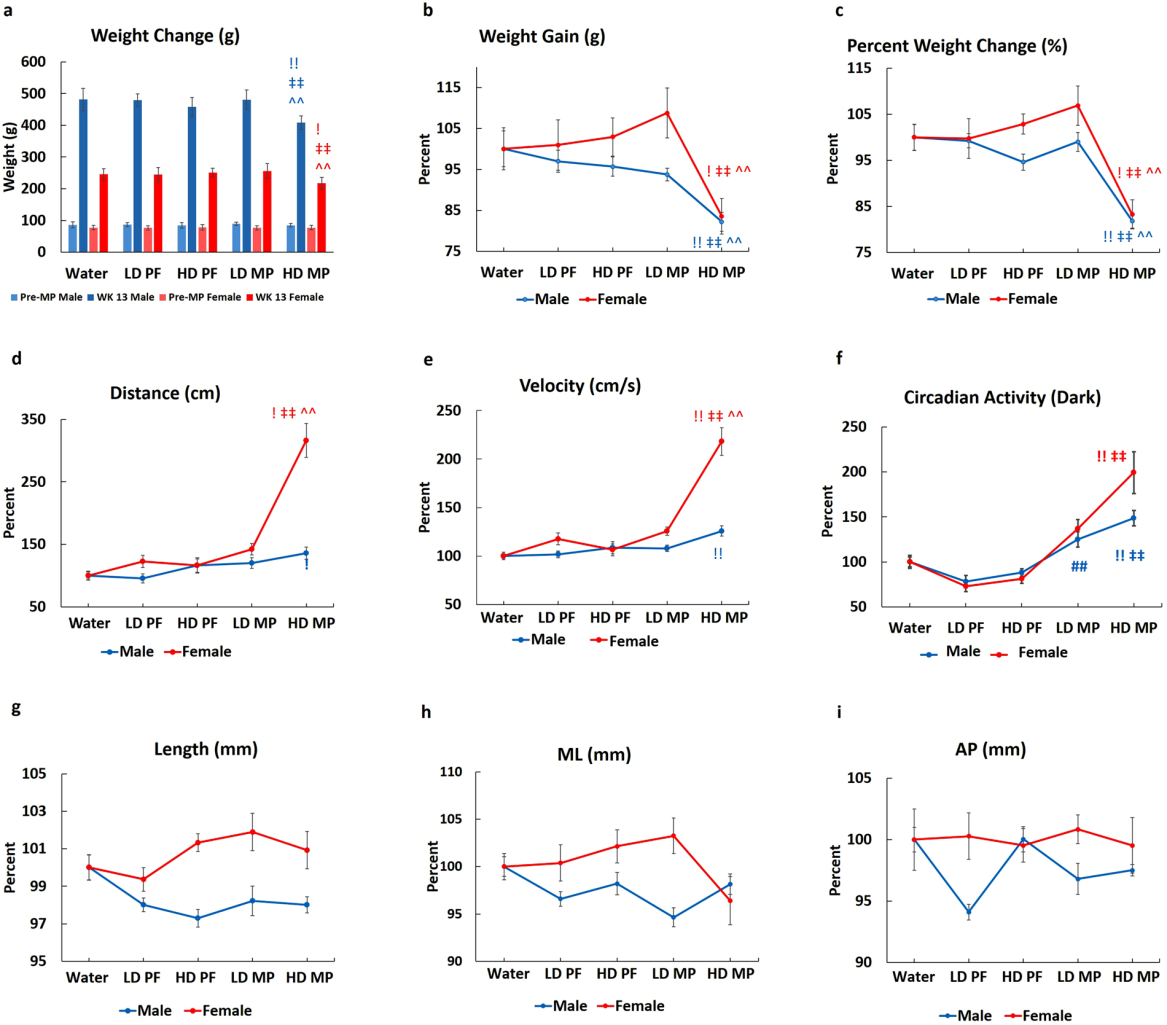
Effects of MP on weight gain and activity: (**a**,**b**,**c**) HD-MP male and female rats showed a significant decrease in weight gain. After 13 weeks of MP administration, HD MP male rats showed less weight gain relative to water (18%), LD PF (17.5%), HD PF (13.5%) and LD (17%) rats. Female rats showed a similar trend, with significantly less weight gain in HD MP female rats (~18%) relative to Water. MP-treated male and female rats also showed increased (**d**) distance traveled, (**e**) velocity, and (**f**) dark circadian activity. MP-treated females covered more distance, with increased velocity, relative to MP-treated male rats (**d**,**e**). HD MP increased circadian activity with an elevated increase in female, as compared to male rats (**f**). MP exposure did not affect femoral (**g**) length, (**h**) medial-lateral diameter, or (**i**) anterior-posterior diameter in male and female rats. (^!^p < 0.05 Water vs HD, ^!!^p < 0.01 Water vs HD, ^‡^p < 0.05 HD PF vs HD, ^‡‡^p < 0.01 HD PF vs HD, ^^^^p < 0.01 LD vs HD, ^##^p < 0.01 LD PF vs LD).

Administration of MP resulted in a dose-dependent reduction in weight gain in male and female rats. No differences in weight gain were observed between Water (Control), LD PF (Pair-fed with Low Dose MP), HD PF (Pair-fed with High Dose MP), and LD MP (Low Dose MP), but HD MP (High Dose MP) males and females gained less weight than Water (Fig. 1a,b,c). HD MP male rats showed less weight gain relative to water (18%), LD PF (17.5%), HD PF (13.5%), and LD (17%) rats. Female rats showed a similar trend, with significantly less weight gain in HD MP female rats (~18%) relative to Water. Activity analyses showed that male and female HD MP rats covered more distance at a higher velocity relative to Water and PF controls (Fig. 1d,e). LD MP rats showed limited increases in distance traveled and velocity (Fig. 1d,e). Female rats treated with MP had a greater increase in activity than males. HD MP females covered ~300% more distance with ~200% higher velocity relative to female Water, while HD MP males covered 35% more distance with 26% higher velocity relative to Water (Fig. 1d,e). Similarly, dark cycle circadian activity showed sex-specific effects as it significantly increased in by ~200% in HD MP females, but only ~48% in HD MP males (Fig. 1f). The effects of MP on skeletal growth were assessed by measuring femoral length, and ML and AP diameters, and no differences were identified (Fig. 1g,h,i).

### MP has sex-dependent effects on bone quality

**Figure 2:**
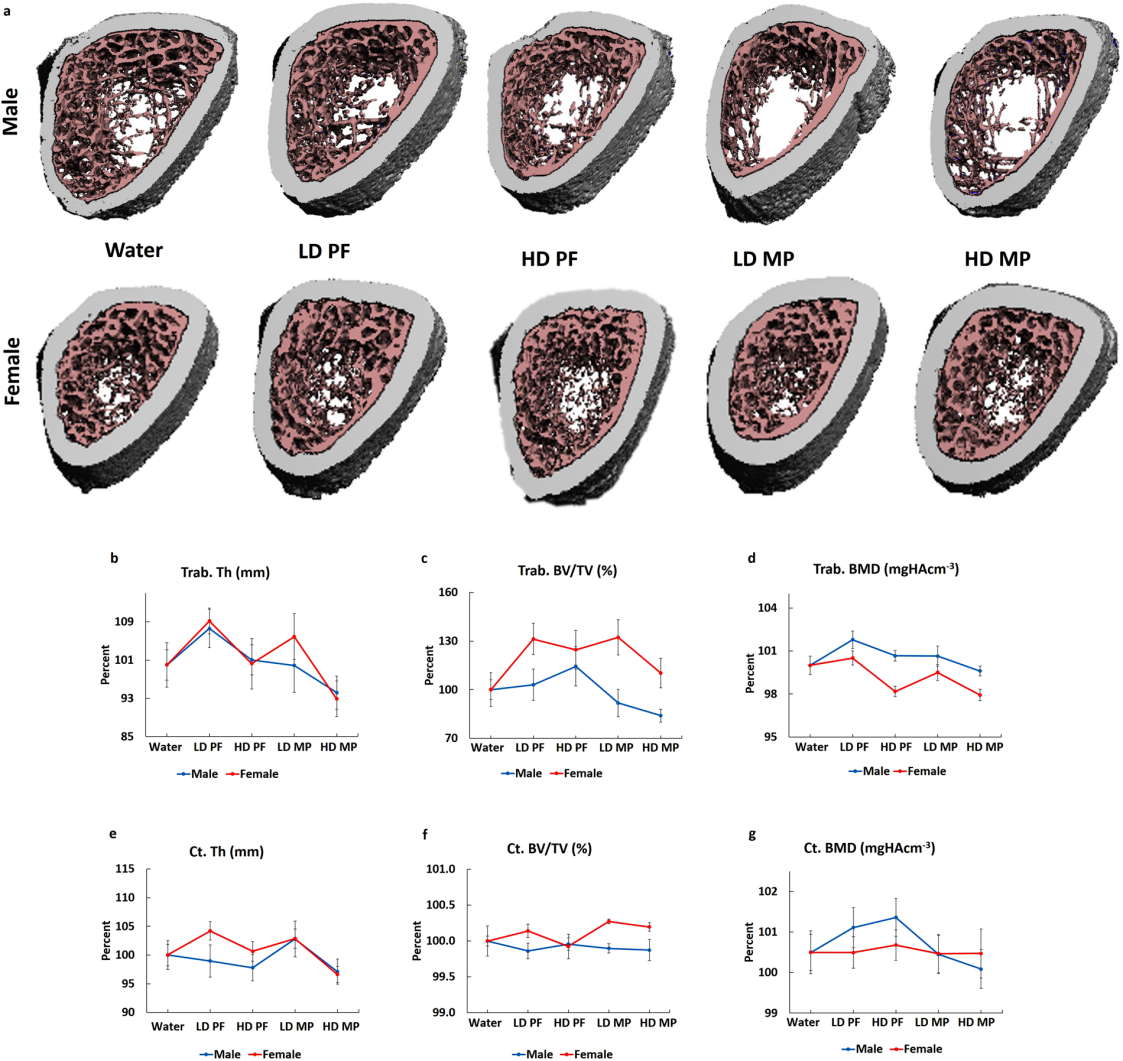
Effects of MP on bone quantity and quality: (**a**) MicroCT scans reveal an apparent decreasing trend in the quantity of trabecular bone in both male and female LD MP and HD MP rats. However, no significant differences were observed in (**b**,**c**,**d**) trabecular and (**e**,**f**,**g**) cortical bone parameters.

**Figure 3:**
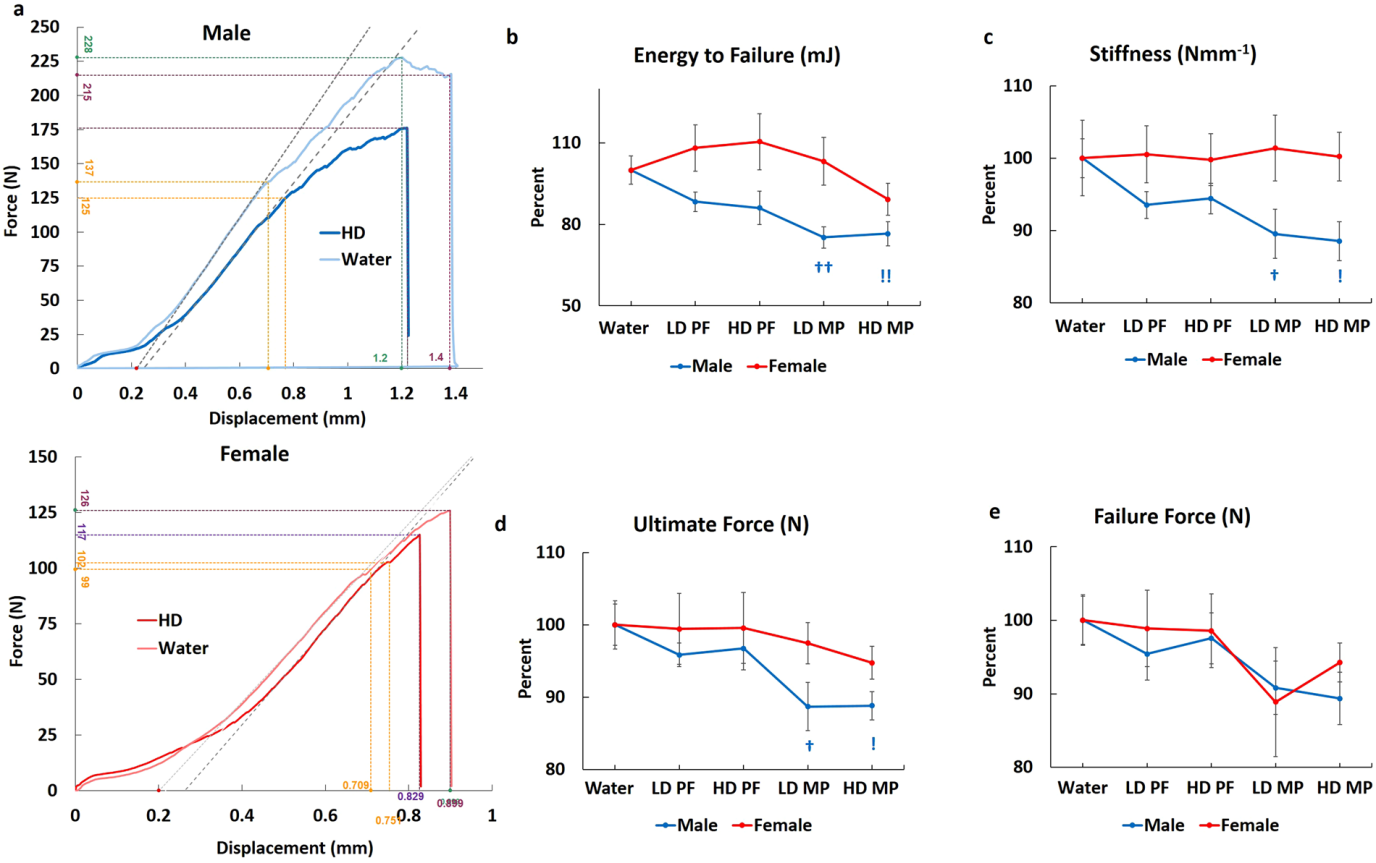
Effects of MP on bone mechanical integrity: (**a**) A right shift was observed in the force-displacement curves of male and female HD MP rats. Male LD and HD MP rats showed a significant decrease in (**b**) energy to failure, (**c**) stiffness, and (**d**) ultimate force relative to Water rats. No significant effects were observed in MP-treated female rats. (**e**) Failure force did not show significant differences in MP-treated male and female rats. (^!^p < 0.05 Water vs HD, ^!!^p < 0.01 Water vs HD, ^‡^p < 0.05 HD PF vs HD, ^‡‡^p < 0.01 HD PF vs HD).

Male and female rat femora were scanned at the metaphysis and mid-diaphysis to assess trabecular and cortical bone quality, respectively. 3D reconstructions show a decreasing trend in trabecular bone with no apparent changes in cortical bone (Fig. 2a). However, quantification showed no significant differences in trabecular thickness (Trab. Th) (Fig. 2b), trabecular bone fraction (Trab. BV/TV) (Fig. 2c), cortical thickness (Ct. Th) (Fig. 2e), cortical bone volume fraction (Ct. BV/TV) (Fig. 2f), or cortical bone mineral density (Ct. BMD) (Fig. 2g) in male and female rats. Trabecular bone mineral density (Trab. BMD) (Fig. 2d) did not show any differeince in male and female rates. (Table S1). Vertebral (L5) microCT analyses also revealed no significant differences in trabecular or cortical bone (Table S2). Three-point bending was performed on femora to determine the effects of MP on biomechanical integrity. HD MP male and female femora showed a right shift on force-displacement curves indicating biomechanical impairment compared to Water (Fig. 3a). HD MP males showed decreased energy to failure, stiffness, ultimate force, and failure force relative to Water (Fig. 3b,c,d,e). In contrast, MP treated females showed no biomechanical impairment (Fig. 3b,c,d,e). Compression testing of L5 vertebra did not show any significant changes in biomechanical parameters MP (data not shown).

### MP mediates bone remodeling

**Figure 4:**
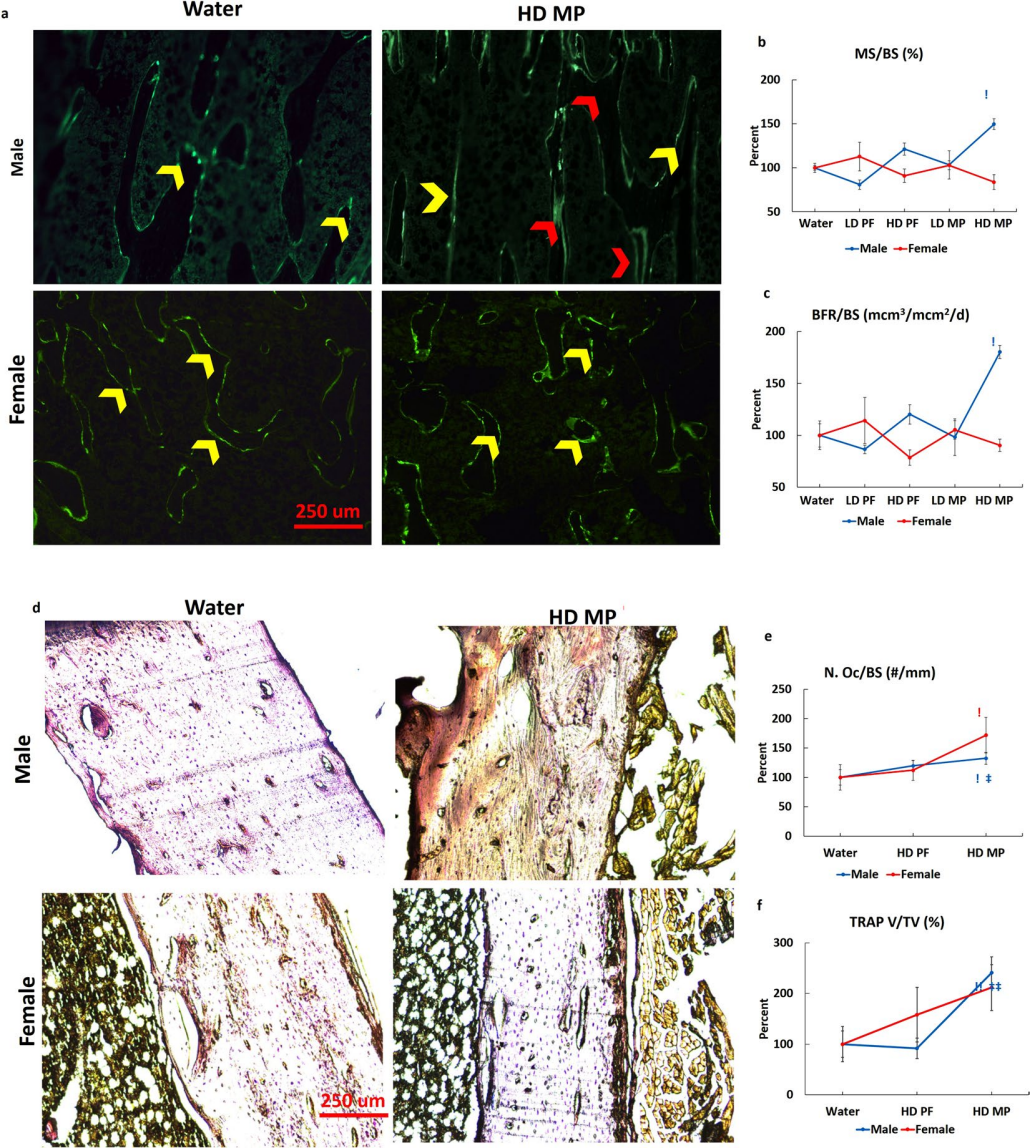
Effects of MP on Bone remodeling: (**a**) No apparent differences in calcein single and double labeling (yellow and red arrows, respectively) were observed in male Water, LD PF, HD PF, and LD MP groups, though male HD MP showed increased calcein labeling. No apparent differences in calcein labeling were seen in female rats. Histomorphometric quantification showed significantly increased (**b**) mineralized bone surface (MS/BS) and (**c**) bone formation rate (BFR/BS) in HD MP male rats with no significant changes in female rats. (**d**) TRAP activity was measured to assess osteoclast activity in cortical bone. HD MP male rats showed increased TRAP staining in cortical bone relative to Water-treated male rats, whereas female rats did not show any apparent differences between Water and HD MP. Quantification of TRAP staining showed a significant increase in (**e**) number of osteoclast over bone surface (N. Oc/BS) in both HD MP treated male and female rats. HD MP treated male rats showed a significant increase in (**f**) TRAP volume over tissue volume (TRAP V/TV) in cortical bone. (^!^p < 0.05 Water vs HD, ^!!^p < 0.01 Water vs HD, ^‡^p < 0.05 HD-PF vs HD, ^‡‡^p < 0.01 HD-PF vs HD).

Dynamic histomorphometric analyses were conducted to evaluate bone formation. Calcein labeling showed increased single and double labeling (Fig. 4a, yellow and red arrows, respectively) in trabecular bone from HD MP males relative to Water, with no apparent differences seen in HD MP females. Quantitative analyses supported these observations with significant increases in mineralized surface and bone formation rate in HD MP males, with no differences seen in females (Fig. 4b,c). Evaluation of cortical parameters found no differences in males or females. TRAP staining was performed to assess bone resorption. HD MP male rats showed increased TRAP staining relative to Water and PF (Fig. 4d). Quantification of TRAP staining confirmed these observations, with significant increases number of osteoclasts over bone surface (1/mm, N. Oc/BS), and TRAP volume fraction (%, TRAP V/TV) (Fig. 4e,f) found for HD MP males. HD MP females also displayed significantly increased osteoclast surface fraction, number, and number of osteoclasts over bone surface (Fig. 4e), but not TRAP volume fraction (Fig. 4f).

### Sex- and dose-dependent effects of MP on osteoclast differentiation and activity ***ex vivo***

**Figure 5:**
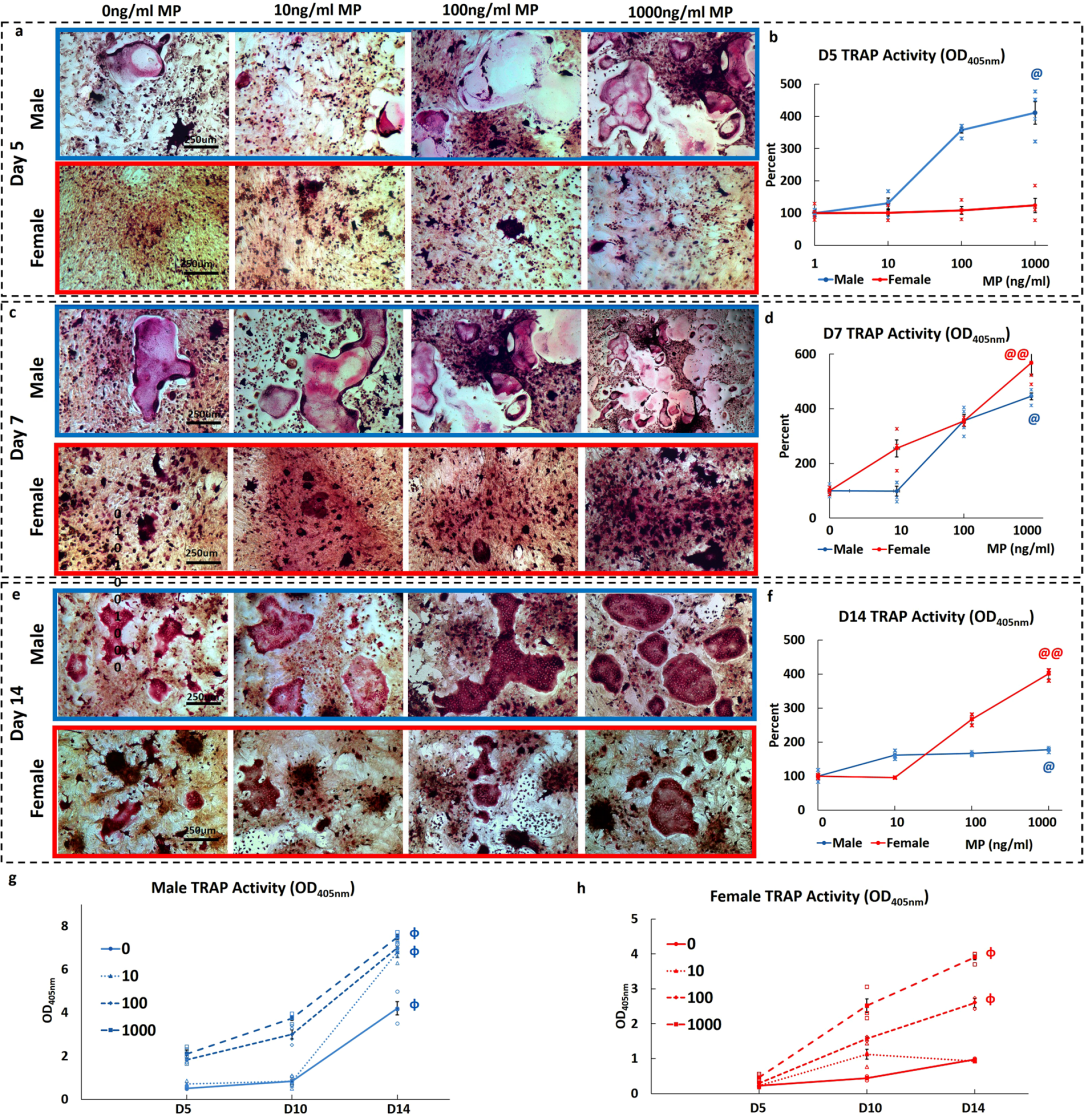
Dose- and sex-dependent effects of MP on osteoclast differentiation and activity: (**a**,**c**, **e**) TRAP staining shows that MP increased osteoclastic differentiation in male and female bone marrow-derived preosteoclasts in a dose- and sex-dependent manner. Males showed the formation of large osteoclasts at day (**a**) 5 that continued to mature at days (**c**) 7, and (**e**) 14. At all time-points, male osteoclasts showed a dose-dependent increase in the number of large osteoclasts with increasing MP concentration. Female osteoclasts also showed accelerated osteoclastogenesis in response to increasing MP concentration but were significantly slower than male cells. Day 5 female osteoclast cultures showed TRAP+ cells clusters, with no large osteoclasts. The number of TRAP+ cell clusters increased at low MP doses and some larger osteoclasts were present at high MP doses at day 7. At day 14, female osteoclasts showed the more typical morphology of mature osteoclast with increasing size and number seen in higher MP doses. (**b**,**d**,**f**) Male and female osteoclasts both showed a dose- and sex-dependent increase in activity, with female osteoclast activity significantly less than male at days 5, 7, and 14. (**g**,**h**) Osteoclast activity as a function of time showed significant increases in osteoclast activity observed between day 5 and 14 for both male and female osteoclasts. (^@^p < 0.05 0 vs 1000 ng/ml, ^@@^p < 0.01 0 vs 1000 ng/ml, ^§§^P < 0.01, male vs female osteoclast activity, ^ɸ^p < 0.05 Day 5 vs Day 7).

**Figure 6:**
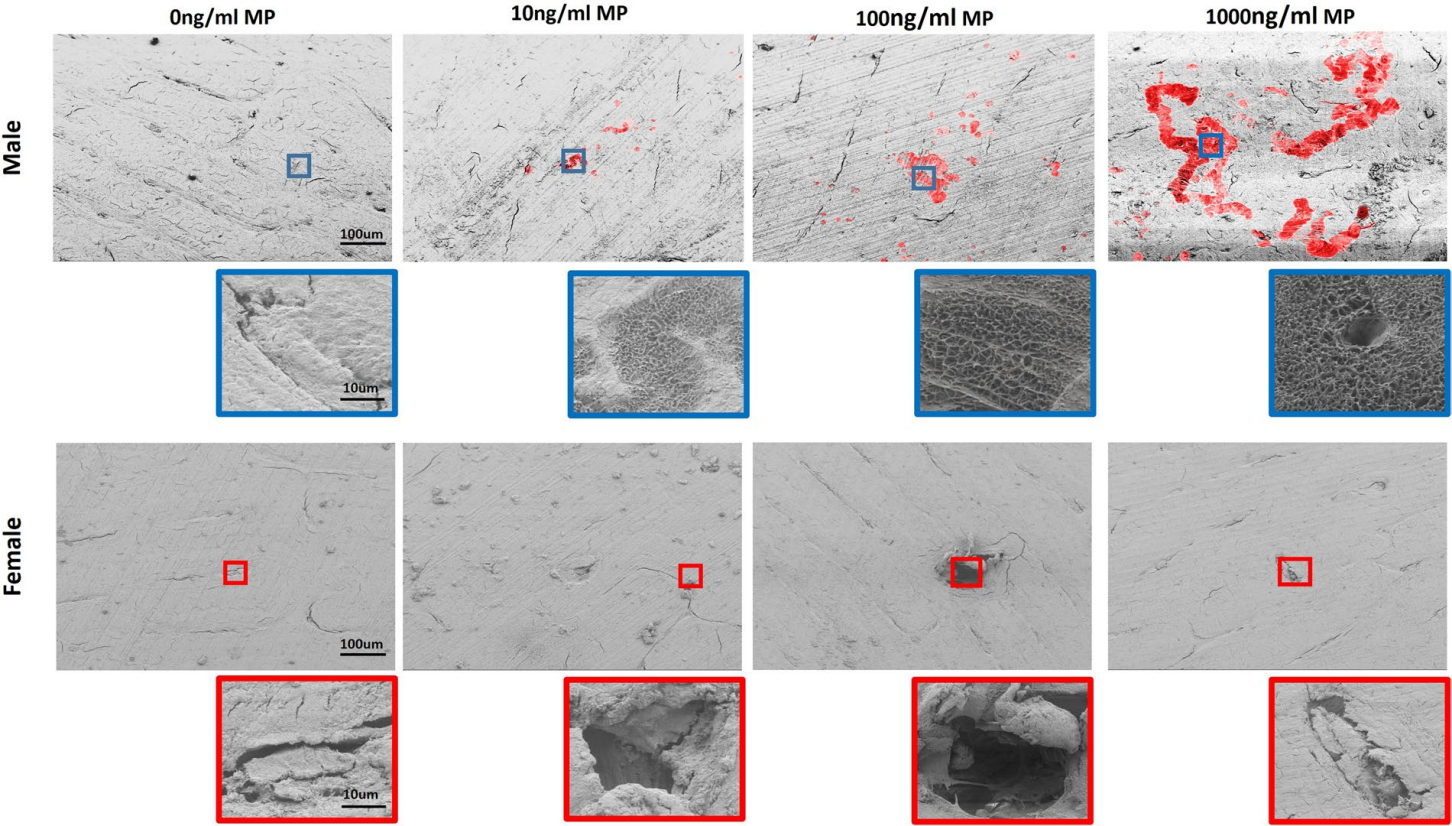
Dose and sex-dependent effects of MP on osteoclast resorption: MP-induced osteoclastic resorption was confirmed using pit assays. SEM analysis at day 18 showed a dose-dependent increase in pit and trail formation (red) in MP-treated male osteoclasts. Both the number of pits and the area covered by pits increased in association with increasing MP doses, with highest pit and trail formation observed at 1000 ng/ml. In contrast, female osteoclasts showed limited resorption, which was mostly limited to mild enlargement of pre-existing cracks. No apparent differences were seen between doses for female osteoclasts.

Male and female preosteoclasts were subjected to osteoclastic induction in the presence of 0, 10, 100, and 1000 ng/ml MP and supplementation with sex-specific rat serum (RS). The resulting osteoclast cultures were stained for TRAP+ cells at days 5, 7, and 14 (Fig. 5a,c,e). Day 5 male cultures showed a dose-dependent increase in the number of large TRAP+ osteoclasts (Fig. 5a). In contrast, female day 5 cultures showed small clusters of TRAP+ cells across MP doses, with no apparent differences (Fig. 5a). Consistent with TRAP staining, day 5 male osteoclasts in 1000 ng/ml MP showed a significant increase in TRAP activity relative to 0 ng/ml (Fig. 5b). Comparison of male and female data showed significantly higher male osteoclast TRAP activity for all MP concentrations, thus indicating higher sensitivity to MP (Fig. 5b). Day 7 male cultures continued to form large TRAP+ osteoclasts, with increased numbers and size at higher doses. Day 7 female cultures also showed a dose-dependent increase in TRAP staining, along with an increasing number of TRAP+ cell clusters, though no large osteoclasts were visible (Fig. 5c). Again, TRAP activity showed a dose-dependent increase in both male and female cultures (Fig. 5d). On day 14, MP-treated male cultures formed large multinucleated TRAP+ cells at all doses, with dose-dependent increases in osteoclast size and number. Female cultures only formed large osteoclasts at higher concentrations (Fig. 5e). TRAP activity assays at day 14 showed significantly higher activity at 1000 ng/ml relative to 0 ng/ml in both male and female cultures (Fig. 5f). In addition, male TRAP activity was significantly higher than female activity at all MP concentrations (Fig. 5f). Evaluating TRAP activity as a function of time showed similar dose-dependent increases in both male and female osteoclast cultures. Significant increases in TRAP activity were recorded in both male and female osteoclasts between day 5 and 14, with higher MP sensitivity seen in male osteoclasts. (Fig. 5g,h). Pit assays were then used to study the resorptive effects of MP by culturing male and female osteoclasts on bovine bone chips for 18 days and analyzing pit and trail formation (pseudocolored red) with SEM (Fig. 6). SEM images showed a dose-dependent increase in pit and trail formation in male osteoclasts, with the highest resorptive activity at 1000 ng/ml MP. In contrast, female osteoclasts showed no evidence of any pit or trail formation, with resorptive activity limited to pre-existing cracks (Fig. 6).

## Discussion

Although the effects of MP on skeletal growth and homeostasis have now been studied for more than 40 years, this is the first study to mechanistically demonstrate that MP has sex- and dose-dependent effects on bone quality via changes in osteoclast activity. Previous studies have shown that MP induced transient growth repression in young growing rats^8^. The current study did not find any significant differences in femoral length and ML and AP diameters in male and female rats. Moreover, histological analyses of the growth plate also failed to identify differences in either sex (data not shown). Weight gains were significantly lower in HD MP male and female rats independent of food intake, as pair-fed rats did not show any significant changes in weight gain over the 13-week study period. Behaviorally, MP administration increased physical activity and intensity in both male and female rats, but female rats covered a higher distance with greater velocity than male rats^11^. Interestingly, the increased physical activity in MP-treated rats did not result in increased osteoblast activity, as shown by dynamic histomorphometry. Osteoblasts are known to be mechanotransductive cells, thus it is possible that MP has a direct or indirect effect on osteoblast activity. Previously, Komatsu *et al*. reported a decrease in BMD and BMC in HD MP male rats^9^. The apparent discrepancy likely arises from the use of different imaging techniques (DXA 2D global imaging vs. microCT 3D localized imaging). Increased physical activity has been shown to promote bone formation, but surprisingly female rats did not show increased bone formation and even showed a small loss in trabecular BMD. Despite limited changes in bone quality, biomechanical integrity was characterized by significant dose- and sex-dependent impairment. MP-treated male rats showed significant dose-dependent decreases in energy to failure, stiffness, ultimate force, and failure force. Similar impairment of biomechanics was not observed in female rats. The discrepancy between bone quality (micro CT analysis) and mechanical integrity can potentially be due to the lower resolution of microCT scans (30 μm). Reduced mechanical strength may have also be caused by degradation of the bone extracellular matrix, particularly collagen structure, which can not be assessed by microCT analysis. TRAP staining provided the first evidence for a cause underlying these sex-dependent effects of MP on cortical bone. HD MP male rats showed a significant increase in TRAP volume fraction, osteoclast number, and osteoclast surface in cortical bone. Similar trends in osteoclast number and surface were observed in females without a concomitant increase in TRAP volume fraction.

The animal studies suggested that the sex- and dose-dependent effects MP on the biomechanical integrity of bone are mediated by altered osteoclastic activity. However, these studies were not able to discern if MP has a direct effect on osteoclasts or if this phenomenon is mediated through secondary pathways. As such, preosteoclasts were differentiated into osteoclasts *ex vivo* and direct MP exposure resulted in increased osteoclastogenesis in both male and female cells and in a dose-dependent manner. The morphology and activity of osteoclasts were also found to differ between males and females. Specifically, male cultures progressively formed large multinucleated osteoclasts through cell fusion. In contrast, female cultures showed the delayed formation of large multinucleated osteoclasts and had a higher number of small TRAP+ cell clusters, indicating delayed cell fusion. MP exposure had dose-dependent effects on male osteoclast maturity, but limited effects on those from females. Additionally, male cultures were more sensitive to the effects of MP. The definitive *ex vivo* data supporting a sex- and dose-dependent effect of MP on osteoclasts is the pit formation assay. Indeed, the results showed a profound dose-dependent elevation of resorptive pits and trails by male osteoclasts, with virtually no resorptive activity seen for female osteoclasts. The *ex vivo* osteoclast experiments confirmed that direct exposure to MP resulted in sex- and dose-dependent effects on osteoclast activity and maturity, consistent with *in vivo* observations. The exact mechanism for the direct action of MP on osteoclasts remains to be determined. It is known that one mechanism of action for MP in the central nervous system is blocking the presynaptic dopamine transporter (DAT) in dopaminergic neurons, thereby increasing the availability of dopamine^12^. However, to date, no studies have identified the presence of DAT in osteoclasts; but interestingly, Bliziotes *et al*. found skeletal biomechanical abnormalities in DAT deficient mice^13^. Additionally, static histomorphometric analyses showed an increase in osteoclast surface fraction in DAT deficient mice. Unfortunately, the study did not evaluate sex-specific effects.

Further studies are required to elucidate the sex-specific interactions between MP and osteoclasts. Although the current study shows that MP has direct effects on osteoclast activity, the data do not preclude the contribution of indirect effects regulated through alterations in osteoblast-mediated RANKL-OPG signaling or through the activity of MP metabolites on the observed skeletal deficiencies. More in-depth analyses of the interactions of MP with osteoclasts, DAT expression, and precise signaling pathways are required to fully understand the effects of MP on osteoclast activity.

In summary, this study establishes for the first time that differential osteoclast regulation is an underlying cellular mechanism for the observed sex- and dose-dependent effects of MP on rat skeletal development. As Feuer *et al*. have documented similar clinically adverse effects^6^, it is vital to conduct further studies to establish clinical protocols to mitigate the adverse effects of MP on skeletal development and bone health. This will protect patients from potentially elevated risks for fractures, as well as ensure achievement of proper peak bone mass to limit any long-term risk of premature osteopenia or osteoporosis.

## Methods and Materials

### Animal Study

All animal experiments were conducted in accordance with the NIH Guide for the Care and Use of Laboratory Animals and were approved by the Stony Brook University Institutional Animal Care and Use Committee.

Four-week-old male and female, Sprague-Dawley rats were randomly distributed into 5 groups (n = 12): (1) Water, a control group that received water with no MP, (2) Low-dose pair-fed (LD PF), a control group that received the same amount of food consumed by low-dose animals, (3) High-dose pair-fed (HD PF), a control group that received the same amount of food consumed by high-dose animals, (4) Low-dose (LD MP), a low MP dosage group, and (5) High-dose (HD MP), a high MP dosage group. MP (Sigma-Aldrich, MO) was delivered via a limited water duel dosage paradigm to replicate therapeutic serum levels^9,14^. Briefly, rats were kept in a reverse light/dark cycle. MP treated groups received 4 mg/kg (LD MP) or 30 mg/kg (HD MP) during the first hour and 10 mg/kg (LD MP) or 60 mg/kg (HD MP) for the next seven hours, with no access to water for the remainder of the day^9,11,14^. Pair-fed groups were given the same amount of food as consumed by respective dosage groups. Body weight, food, and water intake were recorded daily. All the outcome analyses were conducted in a blinded manner (blinded ID sample assignment) with the exception of circadian activity and open field test.

### Circadian Activity (n = 12)

Circadian activity in home cages was recorded by an optical beam sensor (Mini Mitter VitalView software, OR) for 24 hours during last week of treatment. The beam breaks were recorded and analyzed for total beam breaks during the dark and light cycles^14,15^.

### Open Field Test (n = 12)

Rat locomotion was measured using an open-field photobeam activity monitoring system (Coulbourm Instruments, PA) for 90 min during the dark cycle. The data were analyzed for floor plane distance traveled and velocity^14,15^.

### Caliper Measurements (n = 12)

To assess femoral growth, femoral length, and anterior-posterior (AP) and medial-lateral (ML) diameters were measured with digital calipers (Mitutoyo, IL). Femoral length was measured from the femoral head to condyles, and AP and ML diameters were measured at the mid-diaphysis^9^.

### Biomechanics (n = 12)

The biomechanical integrity of the bones was determined using three-point bending. Femora were hydrated in phosphate buffer saline (PBS, Sigma) at room temperature (RT) and placed in a custom designed stainless steel loading jig with an outer span of 20 mm. A monotonic load to failure was applied along the AP axis at a rate of 20 mm/min, under displacement control, using an MTESTQuattro materials testing system equipped with a 1000 N load cell (Admet, MA). Load and displacement were sampled at 100 Hz using the MTESTQuattro software package (Version 3.13.01, Admet). Vertebral biomechanics were assessed using isolated L5 vertebral bodies that were tested in uniaxial compression by applying a monotonic load to failure to the cranial surface at a crosshead speed of 5 mm/min using the same instrument and acquisition parameters. Ultimate force, failure force, stiffness, and energy to failure were then calculated from force vs. displacement plots using a set of custom written macros in Excel (Microsoft, WA)^9^.

### Microcomputed Tomography (MicroCT) (n = 12)

The trabecular bone microstructure was assessed at the distal femoral metaphysis using 18 μm resolution (MicroCT 40, Scanco, Switzerland). To be consistent in analyses, an 11 mm region of the distal femur was scanned and microstructure evaluated for a region of interest (ROI) beginning 250 slices above the epiphysis and continuing distally for 115 slices (~2 mm). Automated scripts were used to evaluate trabecular bone fraction (%, BV/TV), trabecular bone mineral density (g/cm^3^, BMD), trabecular number (1/mm, Tb. N), trabecular thickness (mm, Tb. Th), trabecular separation (mm, Tb.Sp), structural model index (SMI), and bone surface/bone volume (mm^2^/mm^3^, BS/BV). Cortical bone was assessed at the mid-diaphysis using 36 μm resolution. A total of 102 slices were scanned at the midpoint of the femur and 50 slices (~1.7 mm) were evaluated for cortical thickness (mm, Ct. Th), cortical bone mineral density (mgHA/cm^3^, Ct. BMD), endosteal surface (mm^2^, Endo. S), endosteal volume (mm^3^, Endo. V), periosteal surface (mm^2^, Peri. S), and periosteal volume (mm^3^, Peri. V). Vertebral trabecular and cortical bone were evaluated in L5 vertebral segments using 18 μm resolution. The trabecular bone microstructure was quantified for a cylindrical ROI (0.5 mm diameter X 3.6 mm high) in the vertebral body and the previously described parameters were calculated. Vertebral cortical bone was quantified by manual drawing contour lines around the cortical bone for a 3.6 mm high ROI and calculating the previously described parameters^16^.

### Dynamic Histomorphometry (n = 6)

Rats were intraperitoneally injected with 5 mg/kg calcein (Sigma) 10 and 3 days prior to euthanization. Immediately after euthanization, the left tibiae were fixed in 10% formalin (Sigma) for 24 hours and then embedded in polymethyl methacrylate resin (PMMA). Briefly, the bones were serially dehydrated using 70%, 90%, and 100% isopropyl alcohol (Sigma), defatted with acetone/petroleum ether (Sigma) and processed for embedding with three sequential infiltrations of methyl methacrylate (Sigma) and benzoyl peroxide (Sigma). PMMA blocks were polished at 5 μm, coronal metaphyseal and transverse diaphyseal sections were then cut and analyzed for trabecular and cortical bone parameters, respectively. Images were captured using an inverted phase contrast/fluorescent microscope (Nikon, NY) equipped with a digital color (infinity3x, Lumenera, Canada). Trabecular bone was analyzed for mineral deposition rate (μm/day, MAR), mineralized surface fraction (%, MS/BS), and bone formation rate (μm/day, BFR/BS) using BioQuant imaging software (BioQuant, TN). Cortical bone was analyzed for endosteal and periosteal mineralization surface, MS/BS, and BFR, also using BioQuant.

### ***Ex Vivo*** Trap Staining (n = 4)

Right tibiae were decalcified in EDTA-HCl solution (Decal, StatLab), fixed in 10% formalin, and embedded in paraffin. Coronal 5 μm sections were then cut and stained with a tartrate-resistant acid phosphatase (TRAP) kit (Sigma), per the manufacturer’s instructions, to label osteoclasts. Osteoclast surface fraction (% Oc. S/BS), the number of osteoclasts (N. Oc), the number of osteoclasts over the bone surface (1/mm, N. Oc/BS), and TRAP volume over tissue volume (%, TRAP V/TV) were quantified using BioQuant.

### Cell Culture

Bone marrow was flushed from 5-week-old male and female rat long bones. Cells were cultured in DMEM (Gibco, MA) media supplemented with 10% FBS (Hyclone). Cells were allowed to adhere to the surface for 24 hrs in a 37 °C, 5% CO_2_, and humidified atmosphere. Non-adherent cells were collected after 24 hrs and recultured in α-MEM (Gibco) supplemented 10% FBS and 30 ng/ml macrophage colony stimulating factor (MSCF, Abcam, MA). Adherent cells were allowed to proliferate to 70% confluency and media were changed every two days. Preosteoclasts were cultured in α-MEM supplemented with MCSF (30 ng/ml), receptor activator of nuclear factor kappa-B ligand (RANKL, 30 ng/ml, Abcam), and 5% sex-specific rat serum (i.e., male cells in male serum and female cells in female serum). Dose-dependent responses to MP were analyzed by treating cells with 0, 10, 100, or 1000 ng/ml (n = 4, 4000 cells per well) supplemented 5% sex-specific rat serum. Cultures were then analyzed for TRAP+ osteoclasts and activity.

### ***In Vitro*** TRAP Staining (n = 4)

Cells were fixed with 10% formalin for 5 min and stained with a TRAP kit (Sigma) per manufacturer instructions. Cells were analyzed at days 5, 7, and 14 after induction. Cells were imaged under brightfield illumination at 10× with an inverted microscope (Nikon, NY). Two fields of view per well were used to analyze TRAP staining.

### TRAP Activity Quantification (n = 4)

TRAP activity was assessed by using p-nitrophenyl phosphate (*p*NPP, Sigma) as a substrate^17^. Briefly, cells were permeabilized with 0.1% Triton X-100 (Sigma) and the cellular extracts were incubated with pNPP substrate and 0.09 M citrate for 1hr at 37 °C. TRAP activity was then quantified at OD_405nm_ (Spectra iMax3, Molecular Devices, CA).

### Pit Assay (n = 3)

Male and female bone marrow-derived preosteoclasts were cultured in sex-specific rat serum on bovine bone slices (Immunodiagnostic Systems, MD) for 18 days. The slices were decellularized in trypsin-EDTA (Gibco) for 30 min at 37 °C and lysed with 1% Triton X-100 for 15 min at 25 °C. Slices were fixed in 2.5% glutaraldehyde (Sigma) and post-fixed in 1% osmium tetroxide (Sigma). Bone slices were dehydrated with sequential ethanol dilutions and air-dried overnight. Slices were processed for scanning electron microscopy (SEM) and examined at 20 KeV by SEM (Zeiss LEO1550, Germany). The images were pseudocolored (red) using Photoshop (Adobe, CA) to highlight regions of osteoclast trail and pit formation.

### Data analysis and statistics

*In vivo* data were normalized to water treated male and female groups in order to analyze the effects of MP independent of skeletal sexual dimorphism.

Statistical analyses were conducted to calculate significant differences within groups using IBM SPSSS statistics Ver. 23 (IBM), a statistical difference was considered significant for values of p < 0.05. Statistical tests were selected for each analysis with respect to data normality. Circadian, open field, caliper, microCT and biomechanics data were analyzed by one-way ANOVA with posthoc Tukey tests. Histomorphometry, TRAP staining, and *ex vivo* cell culture data were analyzed using Kruskal-Wallis tests.

## Electronic supplementary material

Table S1 and S2
