# Supplementary material for: Methylphenidate regulation of osteoclasts in a dose- and sex-dependent manner adversely affects skeletal mechanical integrity
Source: Sci Rep. 2018 Jan 24;8:1515. doi: 10.1038/s41598-018-19894-x (PMC5784171; doi:10.1038/s41598-018-19894-x)
Supplement: Supplementary file 1 — Table S1 and S2 [file 41598_2018_19894_MOESM1_ESM.pdf]

**Methylphenidate regulation of osteoclasts in a dose- and sex-dependent manner adversely affects skeletal mechanical integrity**

Sardar MZ Uddin[1], Lisa S Robison[2], Dennis Fricke[3], Evan Chernoff[1], Michael Hadjiargyrou[4], Panayotis Thanos[3], David E Komatsu[1]*

## Table S1 - MicroCT analysis of femoral trabecular and cortical bone

| | | Femoral Trabecular MicroCT | | | | | | | Femoral Cortical MicroCT | | | | | | |
|---|---|---|---|---|---|---|---|---|---|---|---|---|---|---|---|
| | Group | BV/TV (%) | Conn-Dens. ($mm^{-3}$) | SMI | Tb. N ($mm^{-1}$) | Tb. Th (mm) | Tb. Sp (mm) | BMD ($mgHAcm^{-3}$) | BV/TV (%) | Tb. Th ($mm^{-1}$) | BMD ($mgHAcm^{-3}$) | Endo. Vol ($mm^3$) | Endo. S ($mm^2$) | Peri. Vol ($mm^3$) | Peri. S ($mm^2$) |
| Male | Water | 0.1808± 0.0117 | 71.72± 4.77 | 1.88± 0.086 | 2.77± 0.159 | 0.0893± 0.003 | 0.3988± 0.02 | 768.65± 5.11 | 0.99 ± 0.00007 | 0.78 ± 0.015 | 967.68 ± 4.51 | 8.28 ± 0.27 | 14.07 ± 0.23 | 21.98 ± 0.40 | 22.75 ± 0.24 |
| | LD PF | 0.1863± 0.0175 | 60.60 4.20 | 1.86± 0.164 | 2.65± 0.187 | 0.0961± 0.004 | 0.4228± 0.03 | 782.34± 4.71 | 0.99 ± 0.0011 | 0.78 ± 0.023 | 973.59 ± 4.99 | 7.97 ± 0.37 | 13.76 ± 0.31 | 21.29 ± 0.41 | 22.32 ± 0.23 |
| | HD PF | 0.2068± 0.0218 | 75.23 6.48 | 1.67± 0.176 | 3.03± 0.265 | 0.0902± 0.003 | 0.3835± 0.04 | 773.75± 2.77 | 0.99 ± 0.0009 | 0.77 ± 0.018 | 976.02 ± 4.75 | 8.06 ± 0.36 | 13.78 ± 0.31 | 21.40 ± 0.62 | 22.40 ± 0.32 |
| | LD MP | 0.1660± 0.0151 | 66.44 3.74 | 1.89± 0.085 | 2.44± 0.197 | 0.0892± 0.005 | 0.4823± 0.05 | 773.54± 5.45 | 0.99 ± 0.0007 | 0.81 ± 0.025 | 967.18 ± 4.73 | 7.19 ± 0.39 | 13.11 ± 0.38 | 20.51 ± 0.52 | 22.23 ± 0.33 |
| | HD MP | 0.1518± 0.0071 | 69.06 3.42 | 2.17± 0.075 | 2.58± 0.091 | 0.0841± 0.003 | 0.4150± 0.02 | 765.58± 2.63 | 0.99 ± 0.0016 | 0.76 ± 0.018 | 963.68 ± 4.88 | 7.97 ± 0.31 | 13.77 ± 0.2819 | 21.09 ± 0.47 | 22.29 ± 0.2536 |
| Female | Water | 0.2386± 0.02 | 78.25 7.71 | 1.50± 0.152 | 3.41± 0.340 | 0.0921± 0.004 | 0.3571± 0.04 | 783.68± 5.04 | 0.99 ± 0.0022 | 0.63 ± 0.016 | 1008.12± 5.60 | 5.28 ± 0.13 | 11.30 ± 0.1511 | 14.65 ± 0.32 | 18.62 ± 0.22 |
| | LD PF | 0.2951± 0.023 | 89.98 7.34 | 1.19± 0.188 | 4.17± 0.346 | 0.1005± 0.002 | 0.2781± 0.03 | 787.63± 3.82 | 0.99 ± 0.0010 | 0.66 ± 0.010 | 1008.05 ± 4.10 | 5.48 ± 0.23 | 11.44 ± 0.25 | 15.35 ± 0.48 | 18.98 ± 0.30 |
| | HD PF | 0.2599± 0.03 | 94.70 6.67 | 1.40± 0.248 | 3.99± 0.265 | 0.0923± 0.005 | 0.2788± 0.02 | 769.39± 2.79 | 0.99 ± 0.0018 | 0.64 ± 0.011 | 1009.94± 3.96 | 5.78 ± 0.14 | 11.82 ± 0.17 | 15.50 ± 0.34 | 19.12 ± 0.29 |
| | LD MP | 0.2749± 0.02 | 90.32 7.37 | 1.37± 0.200 | 3.99± 0.268 | 0.0975± 0.004 | 0.2825± 0.02 | 779.76± 4.33 | 0.99 ± 0.0003 | 0.66 ± 0.011 | 1007.8 ± 4.96 | 5.77 ± 0.20 | 11.76 ± 0.21 | 15.73 ± 0.36 | 19.29 ± 0.23 |
| | HD MP | 0.2302± 0.02 | 89.45 6.91 | 1.58± 0.158 | 3.67± 0.282 | 0.0855± 0.003 | 0.3107± 0.03 | 767.44± 3.08 | 0.99 ± 0.0006 | 0.62 ± 0.009 | 1007.84 ± 6.36 | 5.54 ± 0.29 | 11.43 ± 0.32 | 14.77 ± 0.52 | 18.59 ± 0.35 |

**Table S1 - MicroCT analysis of femoral trabecular and cortical bone:** Trabecular and cortical bone were analyzed using microCT at the femoral metaphysis and mid-diaphysis respectively. MicroCT data did not show any significant differences in trabecular and cortical bone parameters, with the exception of a significant decrease in trabecular bone mineral density in HD MP female rats relative to Water rats.

**Table S2 - MicroCT analysis of L5 vertebra:**

| | | Vertebral Trabecular bone | | | | | | | Vertebral Cortical Bone | | | | |
|---|---|---|---|---|---|---|---|---|---|---|---|---|---|
| | **Group** | **BV/TV (%)** | **Conn-Dens. ($mm^{-3}$)** | **SMI** | **Tb. N ($mm^{-1}$)** | **Tb. Th (mm)** | **Tb. Sp (mm)** | **BMD ($mgHAcm^{-3}$)** | **BV/TV (%)** | **Cort. Th ($mm^{-1}$)** | **BMD ($mgHAcm^{-3}$)** | **Peri. V ($mm^3$)** | **Peri. S ($mm^2$)** |
| Male | **Water** | 0.192 ± 0.01 | 44.592 ± 3.45 | 1.547 ± 0.11 | 3.123 ± 0.12 | 0.086 ± 0.003 | 0.350 ± 0.013 | 710.96 ± 3.84 | 0.655 ± 0.03 | 0.136 ± 0.007 | 846.468 ± 5.44 | 0.758 ± 0.043 | 12.195 ± 0.45 |
| | **LD PF** | 0.212 ± 0.01 | 48.723 ± 5.78 | 1.450 ± 0.11 | 3.345 ± 0.16 | 0.092 ± 0.004 | 0.330 ± 0.013 | 718.68 ± 5.02 | 0.657 ± 0.03 | 0.138 ± 0.006 | 847.228 ± 4.31 | 0.724 ± 0.036 | 11.710 ± 0.35 |
| | **HD PF** | 0.212 ± 0.02 | 50.770 ± 3.81 | 1.318 ± 0.20 | 3.066 ± 0.16 | 0.088 ± 0.004 | 0.361 ± 0.017 | 724.92 ± 5.89 | 0.658 ± 0.01 | 0.139 ± 0.004 | 852.605 ± 6.27 | 0.710 ± 0.018 | 11.530 ± 0.20 |
| | **LD MP** | 0.225 ± 0.01 | 64.420 ± 5.77 | 1.397 ± 0.08 | 3.686 ± 0.21 | 0.086 ± 0.002 | 0.301 ± 0.018 | 715.97 ± 3.17 | 0.673 ± 0.01 | 0.143 ± 0.004 | 853.127 ± 3.12 | 0.740 ± 0.028 | 11.770 ± 0.28 |
| | **HD MP** | 0.172 ± 0.01 | 51.326 ± 5.54 | 1.708 ± 0.06 | 2.755 ± 0.14 | 0.084 ± 0.001 | 0.403 ± 0.021 | 715.69 ± 3.84 | 0.669 ± 0.01 | 0.138 ± 0.003 | 845.664 ± 3.23 | 0.737 ± 0.029 | 11.979 ± 0.32 |
| Female | **Water** | 0.275 ± 0.01 | 57.729 ± 7.21 | 0.793 ± 0.12 | 3.557 ± 0.14 | 0.097 ± 0.002 | 0.311 ± 0.014 | 730.71 ± 3.22 | 0.703 ± 0.01 | 0.148 ± 0.004 | 877.885 ± 6.35 | 0.637 ± 0.021 | 10.022 ± 0.23 |
| | **LD PF** | 0.263 ± 0.02 | 72.881 ± 12.06 | 0.745 ± 0.26 | 3.543 ± 0.28 | 0.092 ± 0.003 | 0.328 ± 0.023 | 715.75 ± 6.42 | 0.679 ± 0.01 | 0.145 ± 0.003 | 863.917 ± 6.66 | 0.614 ± 0.013 | 9.807 ± 0.15 |
| | **HD PF** | 0.301 ± 0.01 | 64.314 ± 6.26 | 0.454 ± 0.10 | 3.651 ± 0.15 | 0.099 ± 0.002 | 0.310 ± 0.01 | 732.39 ± 3.24 | 0.678 ± 0.01 | 0.140 ± 0.004 | 869.212 ± 3.85 | 0.618 ± 0.020 | 9.907 ± 0.21 |
| | **LD MP** | 0.277 ± 0.02 | 62.809 ± 7.76 | 0.632 ± 0.19 | 3.431 ± 0.15 | 0.094 ± 0.003 | 0.327 ± 0.014 | 715.87 ± 6.25 | 0.702 ± 0.01 | 0.150 ± 0.004 | 875.367 ± 8.52 | 0.657 ± 0.023 | 10.239 ± 0.24 |
| | **HD MP** | 0.260 ± 0.01 | 52.318 ± 4.22 | 0.727 ± 0.09 | 3.282 ± 0.16 | 0.092 ± 0.001 | 0.337 ± 0.016 | 718.08 ± 3.45 | 0.671 ± 0.01 | 0.140 ± 0.003 | 856.564 ± 4.77 | 0.618 ± 0.018 | 9.917 ± 0.18 |

**Table S2 - MicroCT analysis of L5 vertebra:** Trabecular and cortical bone quantity in L5 vertebra were assessed using MicroCT. No significant changes were observed in male and female rats relative to MP treatments.
